# Variation in Virus Symptom Development and Root Architecture Attributes at the Onset of Storage Root Initiation in ‘Beauregard’ Sweetpotato Plants Grown with or without Nitrogen

**DOI:** 10.1371/journal.pone.0107384

**Published:** 2014-09-22

**Authors:** Arthur Q. Villordon, Christopher A. Clark

**Affiliations:** 1 Sweet Potato Research Station, Louisiana State University Agricultural Center, Chase, Louisiana, United States of America; 2 Department of Plant Pathology and Crop Physiology, Louisiana State University Agricultural Center, Baton Rouge, Louisiana, United States of America; Virginia Tech, United States of America

## Abstract

It has been shown that virus infections, often symptomless, significantly limit sweetpotato productivity, especially in regions characterized by low input agricultural systems. In sweetpotatoes, the successful emergence and development of lateral roots (LRs), the main determinant of root architecture, determines the competency of adventitious roots to undergo storage root initiation. This study aimed to investigate the effect of some plant viruses on root architecture attributes during the onset of storage root initiation in ‘Beauregard’ sweetpotatoes that were grown with or without the presence of nitrogen. In two replicate experiments, virus-tested plants consistently failed to show visible symptoms at 20 days regardless of nitrogen treatment. In both experiments, the severity of symptom development among infected plants ranged from 25 to 118% when compared to the controls (virus tested plants grown in the presence of nitrogen). The presence of a complex of viruses (*Sweet potato feathery mottle virus*, *Sweet potato virus G*, *Sweet potato virus C*, and *Sweet potato virus 2*) was associated with 51% reduction in adventitious root number among plants grown without nitrogen. The effect of virus treatments on first order LR development depended on the presence or absence of nitrogen. In the presence of nitrogen, only plants infected with *Sweet potato chlorotic stunt virus* showed reductions in first order LR length, number, and density, which were decreased by 33%, 12%, and 11%, respectively, when compared to the controls. In the absence of nitrogen, virus tested and infected plants manifested significant reductions for all first order LR attributes. These results provide evidence that virus infection directly influences sweetpotato yield potential by reducing both the number of adventitious roots and LR development. These findings provide a framework for understanding how virus infection reduces sweetpotato yield and could lead to the development of novel strategies to mitigate virus effects on sweetpotato productivity.

## Introduction

Sweetpotato (*Ipomoea batatas*) is propagated vegetatively and in the process, propagating material gradually accumulates pathogens, especially viruses that cause decline in yield and quality. At least 30 different viruses have been isolated from sweetpotato [1], but only a few of these consistently cause problems in sweetpotato production. *Sweet potato chlorotic stunt virus* (SPCSV) is a phloem-restricted, whitefly-transmitted virus that is widespread in Africa and South America. SPCSV by itself causes significant yield reduction, but also represses resistance in sweetpotato to other viruses leading to synergistic interactions that reduce yields 80–90% [1], [2]. In the U.S., sweetpotatoes are universally infected by a complex of four aphid-transmitted potyviruses: *Sweet potato feathery mottle virus* (SPFMV), *Sweet potato virus C* (SPVC), *Sweet potato virus G* (SPVG), and *Sweet potato virus 2* (SPV2) [1]. These potyviruses infect most tissues within plants and the complex can cause yield losses of 25–40% under some circumstances [1], [3]. However, it is not yet clear if this yield reduction is due to virus effects on storage root initiation, on subsequent storage root bulking, or both.

In sweetpotatoes, the most economically important physiological process is storage root initiation, defined as the appearance of cambia around the protoxylem and secondary xylem elements [4], [5], [6], [7]. Prior work, utilizing diagnostic anatomical features, has defined key stages of sweetpotato storage root yield determination: early “tuberous-root” thickening or storage root initiation stage (up to 25 days after transplanting, DAT), “middle stage” (25–60 DAT), and the “late stage” (from 60 DAT to harvest) [5]. Recently, it has been shown that lateral root development, a key determinant of root system architecture, is fundamentally associated with the competency of adventitious roots to undergo storage root initiation [8]. Root system architecture has been referred to as an integrative result of LR initiation, morphogenesis, emergence, and growth [9]. LRs contribute to water-use efficiency and facilitate the extraction of micro- and macronutrients from the soil [10]. This relationship between root architecture and the developmental fate of adventitious roots addresses the related issues of understanding how the sweetpotato plant modulates storage root initiation and how differential root carbon sink is determined within the root system [11], [12]. Knowledge of the variables that control root architecture development can be integrated with other variables that are known to influence storage root yields, enabling a more systematic approach to determining and managing yield constraints of this globally important root crop.

It has been shown in model systems that intrinsic and environmental variables influence LR development. Internal cues of LR formation include auxin, ethylene, abscisic acid, cytokinin, and strigolactones [13], [14], [15], [16]. External variables include growth substrate water availability [17] and soil nutrients such as ammonium (NH_4_^+^) [18], nitrate (NO_3_^−^) [19], phosphate [20], and sulfate [21]. The roles of growth substrate water status and nitrogen variability in altering root architecture during the onset of storage root initiation have recently been validated in ‘Beauregard’ sweetpotato [8], [22]. Such findings can lead directly to the development and testing of management practices for improved economic yields, water use efficiency and nutrient foraging. Recent work in model systems has demonstrated that virus infections can lead to alterations in lateral root development [22], [23], [24]. These findings provide evidence about the potential influence of biotic factors on root architecture development. The primary objective of this work was to investigate the effect of selected plant viruses on root architecture characteristics as measured by LR development attributes in ‘Beauregard’ sweetpotato plants grown with or without the presence of nitrogen.

## Materials and Methods

### Viruses

Virus-tested (V0) plants of sweetpotato cv. ‘Beauregard’, mericlone B-14 (V0 B-14), were produced originally by meristem-tip culture [3], [30] and tested by three successive grafts to seedlings of the indicator plant *Ipomoea setosa*, by PCR for geminivirus detection as described by Li et al. [27], and by real-time PCR for detection of SPCSV [28] and found to be apparently free of known viruses. V0 plants were maintained by nodal propagation in tissue culture in the Louisiana State University Agricultural Center Foundation Seed Program. To produce plants for different virus treatments, V0 B-14 plants were inoculated separately with each virus treatment (potyvirus complex [V1] and SPCSV [V2]) by grafting with sweetpotato scions infected with the appropriate virus(es) and subsequently maintaining infected plants by periodically transplanting vine cuttings from infected stocks. Two sources of viruses were used in these experiments: one to provide the common U.S. complex of potyviruses and another to provide SPCSV. The potyvirus complex source plant was originally provided by Dr. G. C. Yencho (Dept. Horticultural Sciences, North Carolina State University, Raleigh). The infected ‘Beauregard’ plant (B-14, G-7) had been grown in the field for seven years during which it was naturally infected. Clone B-14, G-7 was tested by grafting to *I. setosa* and testing the symptomatic *I. setosa* by ELISA on nitrocellulose membranes (NCM-ELISA) using antisera provided by S. Fuentes (International Potato Center, Lima, Peru), multiplex PCR for potyviruses [26], PCR for geminiviruses [27], and real-time RT-PCR [28] and found to be infected with SPFMV, SPVC, SPVG, and SPV2 but tested negative for *Sweet potato mild mottle virus*, *Sweet potato latent virus*, *Sweet potato chlorotic fleck virus*, *Sweet potato mild speckling virus*, *Sweet potato leaf curl virus* and other related geminiviruses, *Sweet potato chlorotic stunt virus* (SPCSV), *Sweet potato collusive virus*, and *Cucumber mosaic virus* (CMV). However, the possibility that it was infected by viruses not yet recognized in sweetpotato cannot be eliminated. ‘Beauregard’ plants infected with the U.S. strain of SPCSV, isolate BWFT-3 which was initially obtained by single-whitefly transmission [28], was maintained by nodal propagation in tissue culture. The V1 and V2 plants were maintained and increased by propagating infected vine cuttings and their associated vegetatively propagated viruses.

### Source plants

Source plants for cuttings for experiments were produced in a greenhouse at the Louisiana State University Agricultural Center, Baton Rouge (30.411380 N, 91.172807 W). A rigorous program of insecticide application, sticky card trapping, and sanitation was routinely employed to manage potential insect vectors of viruses. Plants were propagated in 1∶1∶1 river silt: sand: Jiffy Mix Plus (Jiffy Products of America, Inc.) amended with Osmocote 14-14-14 (Scotts-Sierra Horticultural Products Company) at 3.5 gm Kg^−1^ soil mix. Source plants were grown in 32-cell Speedling trays (Speedling, Inc.) until 25–30 cm long terminal vine cuttings could be taken.

### Experimental design and treatments

Experiments were conducted in the same greenhouse. There were two replicate experiments, and the planting dates were 13 November and 19 December 2013. In all experiments, vegetative terminal vine cuttings were selected that were 25–30 cm long, with five to six fully opened leaves, approximately 5 mm diameter at the basal cut, and with uniform distribution of nodes. Cuttings were planted (2–3 nodes under the growth substrate surface) in 10 cm-diameter polyvinyl chloride pots (height = 30 cm) with detachable plastic bottoms. The growth substrate and experimental conditions used in this study were based on previously developed methodology for measuring the effect of growth substrate moisture and nitrogen content on root architecture development at the onset of storage root initiation in ‘Beauregard’ [8], [22]. The growth substrate for all experiments was washed river sand. The diameter of sand particles varied from 0.05 to 0.9 mm with the majority (83%) in the 0.2- to 0.9-mm range. For plants grown in the presence of nitrogen (+N), the nutrient was provided as KNO_3_ at the rate of 50 kg·ha^−1^ while each of P_2_O_5_ and K_2_O was supplied at the rate equivalent to 134 kg·ha^−1^. Similar P_2_O_5_ and K_2_O rates were used for plants grown without nitrogen (-N). The greenhouse temperature regime was 29°C for 14 hr (day) and 18°C for 10 hr (night). Photosynthetic photon flux (PPF) for plant production and subsequent experiments ranged from 300 to 800 m^−2^ s^−1^. High intensity mercury vapor lamps were used to extend daylength to 14 h per day. PPF was measured at the canopy level with a quantum sensor (Model QSO-S, Decagon Devices Inc., Pullman, WA). The relative humidity averaged 60%. Temperature and relative humidity were monitored at the canopy level using an integrated temperature and relative humidity sensor (Model RHT, Decagon Devices Inc.). The moisture of the growing substrate was maintained ≈65 to 75% of field capacity (≈12% volumetric water content). Growth substrate moisture was measured with ECH2O soil moisture sensors (Model EC-5, Decagon Devices Inc.) inserted vertically at the 2–7 cm depth.

During the experiments, plants infected with the potyvirus complex developed typical symptoms of chlorotic spotting followed by development of purple borders around the spots on leaves. Plants infected with SPCSV developed interveinal purple blotches. In both cases, symptoms developed primarily, but not exclusively on the older leaves. Virus symptom severity was assessed the day before each experiment was terminated by rating each leaf by visual estimation of the proportion of the leaf showing symptoms using a 0 to 3 scale in which: 0 =  no symptoms, 1 =  <1/3 of the leaf involved, 2 = 1/3–2/3 of leaf area involved, and 3 = >2/3 of the leaf involved. The mean rating for all leaves on a plant was used for statistical analysis and comparison of treatments.

All experiments were arranged in a randomized complete block design where a pot (1 plant per pot) was considered a replicate. There were four replicates in the first experiment but one replicate was lost prior to data collection. There were five replicates in the second experiment. All experiments were terminated after 20 days by removing the detachable plastic bottoms tilting the pots and removing the growth substrate gradually using a stream of water.

### Root architecture measurements

Data collection followed the procedures described in previous work [8], [22]. Intact adventitious roots that were 20 cm or greater in length were floated on waterproof trays and scanned using a specialized Dual Scan optical scanner (Regent Instruments Inc., Quebec, Canada). Based on previous work, adventitious roots that were less than 20 cm in length generally failed to show anatomical features associated with lignification or storage root initiation [6], [8]. The acquisition and image analysis software was WinRHIZO Pro (v. 2009c; Regent Instruments Inc.). Root types (main root, first order LRs, second order LRs) were automatically classified based on root diameter which was in turn based on predetermined size intervals that were dynamically adjusted between samples. In the present work, three intervals were used: 0 to 0.2, 0.2 to 1.0, and 1.0 to 20 mm. LR attributes that were measured from scanned images included first and second order LR number and length. First order LR density was calculated by dividing first order LR number by the length of the main root.

### Statistical analyses

Root attribute data from each experiment were pooled after verifying the lack of planting date effects. Root length and number were transformed using log 10 and square root transformation, respectively. Statistical analyses were performed using SAS Proc Mixed (SAS v. 9.1, SAS Inc., Cary, NC).). Fisher's LSD test at the 0.05 probability level was used to test for statistical significance. The data presented were means and standard error of the means from non-transformed data.

## Results

### Symptom development

**Figure 1:**
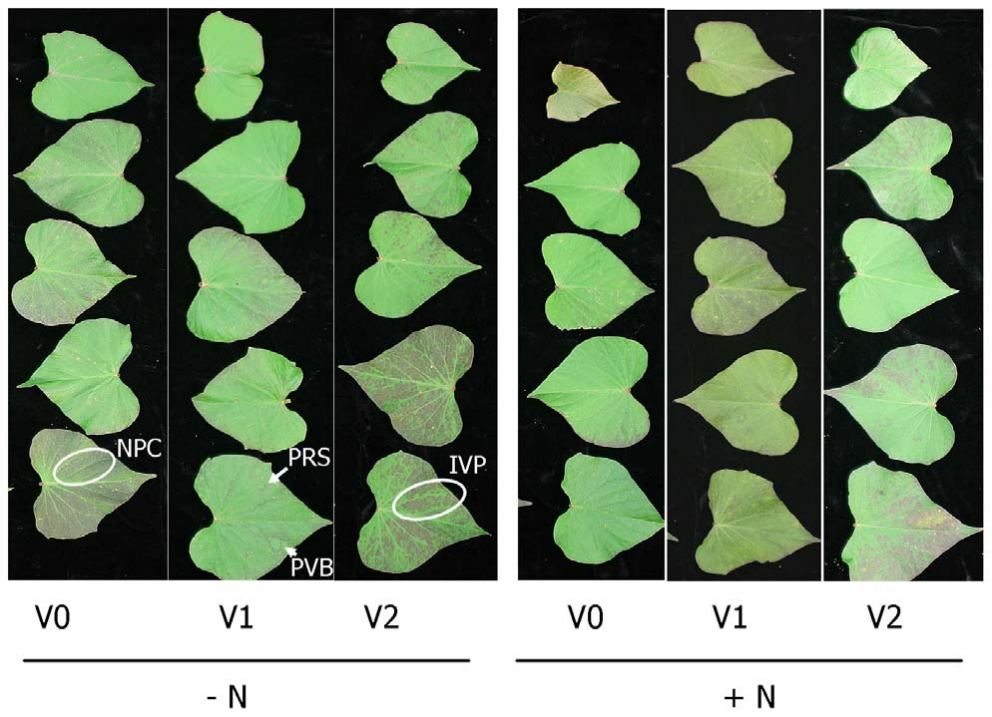
Virus symptoms on ‘Beauregard’ sweetpotato leaves the day before the first experiment was terminated. All expanded leaves originating from nodes above the substrate surface are depicted with the bottom leaf in each column being the oldest and the top being the youngest. + N  =  nitrogen provided as KNO_3_, - N = no nitrogen provided. V0  =  plants derived from non-inoculated, virus-tested plant stock; V1  =  plants derived from V0 plant stock graft inoculated with the potyvirus complex (SPFMV, SPVG, SPVC, and SPV2); V2  =  plants derived from plant stock infected with SPCSV. Potyvirus symptoms consist of purple ring spots (PRS, see arrow) and purple vein banding (PVB arrow). *Sweet potato chlorotic stunt virus* symptoms include deep interveinal purpling (IVP circle) that is distinguished from the natural purple cast (NPC circle) that develops on some sweetpotato leaves in that with IVP veins remain green, and the pigmentation is deeper.

**Figure 2:**
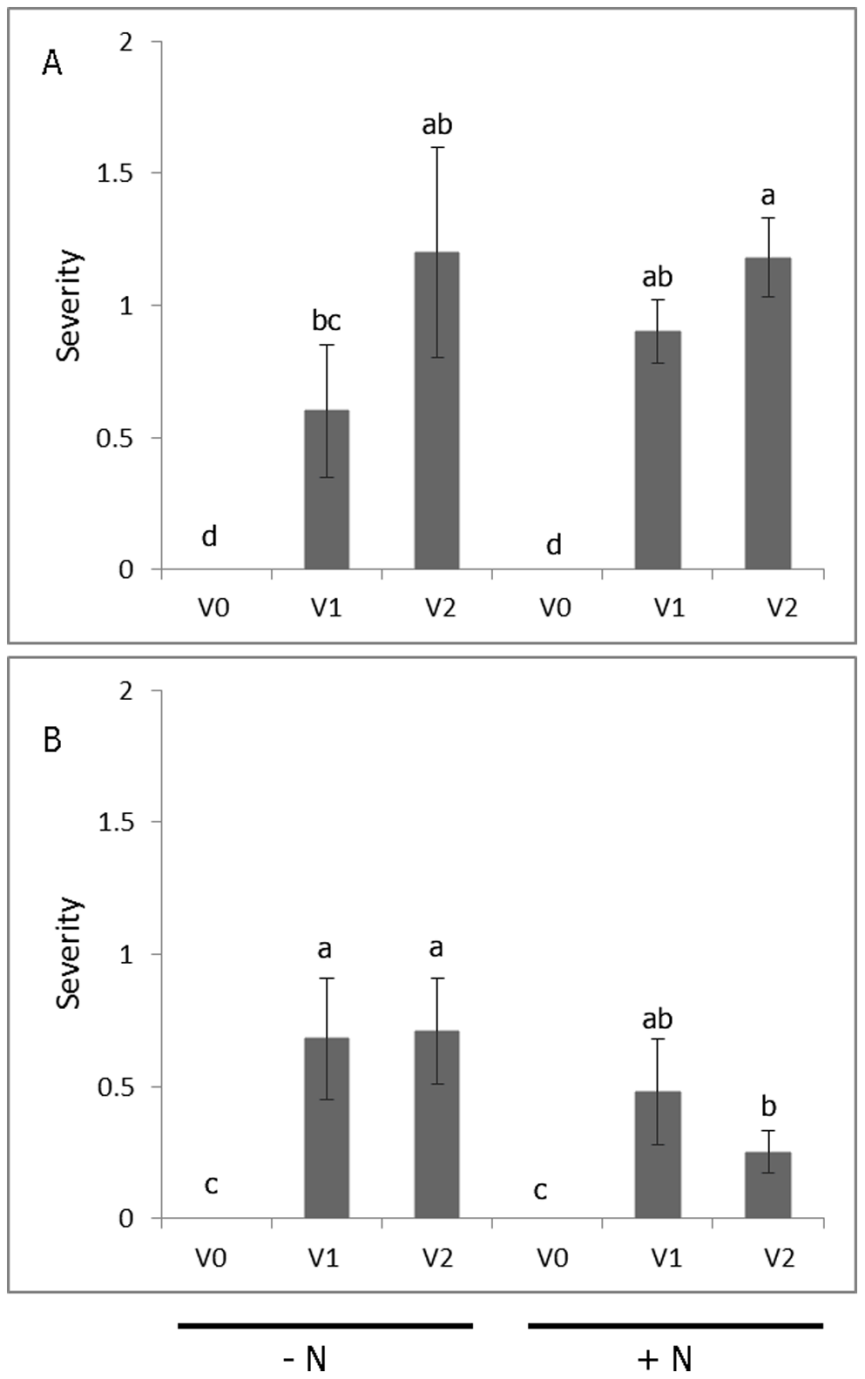
Virus symptom severity ratings of plants from the first (A) and second (B) experiments. Virus symptom severity was assessed the day before each experiment was terminated by rating each leaf by visual estimation of the proportion of the leaf showing symptoms using a 0 to 3 scale in which: 0 =  no symptoms, 1 = <1/3 of the leaf involved, 2 = 1/3–2/3 of leaf area involved, and 3 = >2/3 of the leaf involved. + N  =  nitrogen provided as KNO_3_, - N = no nitrogen provided. V0  =  plants derived from non-inoculated, virus-tested plant stock; V1  =  plants derived from V0 plant stock graft inoculated with the potyvirus complex (SPFMV, SPVG, SPVC, and SPV2); V2  =  plants derived from plant stock infected with SPCSV. Severity ratings were transformed using log 10 and Fisher's LSD test at the 0.05 probability level was used to test for statistical significance. The data are expressed as means ± SE from non-transformed data.

No virus symptoms were observed on the non-inoculated, virus-tested plants at any time during propagation of source plants or during the experiments. Virus symptoms first appeared on the infected plants at about 2 wk after transplanting and became gradually more pronounced until the experiments were terminated at 20 days after transplanting (Figs. 1,2). Virus symptoms develop earlier, are more pronounced and more consistent on plants grown in sand than in other more complex substrates (Clark, unpublished data). Although there were no significant differences in symptom severity between nitrogen treatments, symptom severity was lower in the treatments with nitrogen added in the second test (Fig. 2B).

### Root architecture attributes

**Figure 3:**
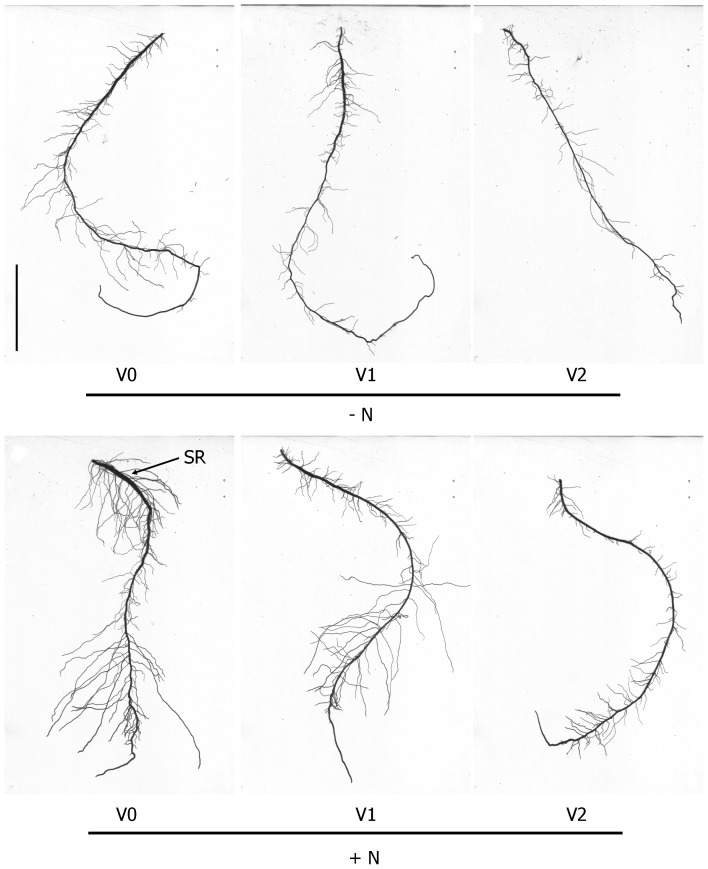
Representative adventitious roots from sweetpotato ‘Beauregard’ plants subjected to different virus treatments and grown with or without nitrogen. + N  =  nitrogen provided as KNO_3_, - N = no nitrogen provided. V0  =  plants derived from non-inoculated, virus-tested plant stock; V1  =  plants derived from V0 plant stock graft inoculated with the potyvirus complex (SPFMV, SPVG, SPVC, and SPV2); V2  =  plants derived from plant stock infected with SPCSV. SR = localized swelling indicative of successful storage root initiation. Scale bar  = 5 cm.

**Figure 4:**
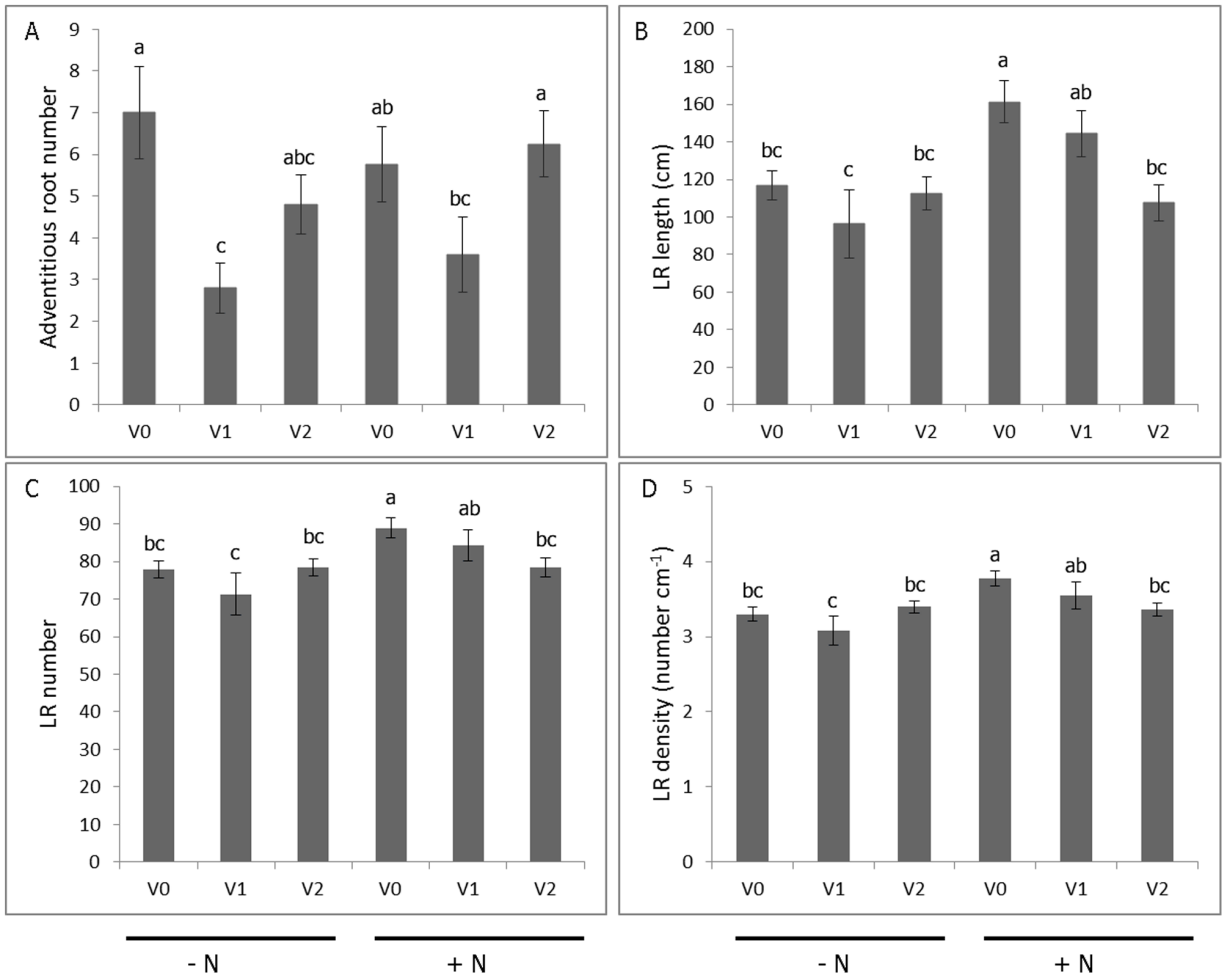
Variation in adventitious root number (A), first order lateral root length (B), first order lateral root number (C), and lateral root density (D) in response to virus and nitrogen treatments at the onset of storage root initiation in ‘Beauregard’ sweetpotato. + N  =  nitrogen provided as KNO_3_, - N = no nitrogen provided. V0  =  plants derived from non-inoculated, virus-tested plant stock; V1  =  plants derived from V0 plant stock graft inoculated with the potyvirus complex (SPFMV, SPVG, SPVC, and SPV2); V2  =  plants derived from plant stock infected with SPCSV. Root length and number were transformed using log 10 and square root transformation, respectively, and Fisher's LSD test at the 0.05 probability level was used to test for statistical significance. The data are expressed as means ± SE from non-transformed data.

**Figure 5:**
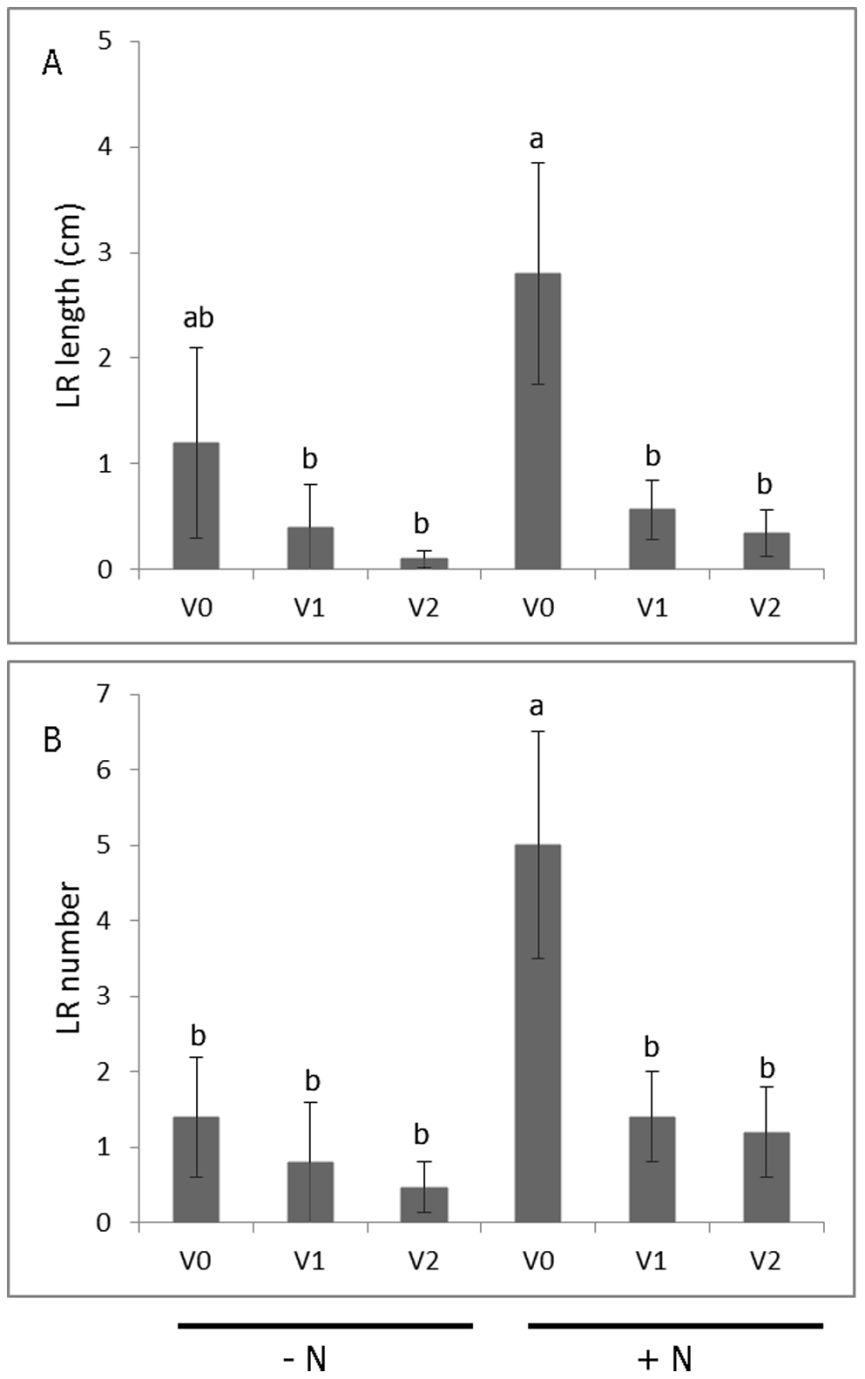
Variation in second order lateral root length (A) and number (B) in response to virus and nitrogen treatments at the onset of storage root initiation in ‘Beauregard’ sweetpotato. Treatment legend: + N  =  nitrogen provided as KNO_3_, - N = no nitrogen provided. V0  =  plants derived from non-inoculated, virus-tested plant stock; V1  =  plants derived from V0 plant stock graft inoculated with the potyvirus complex (SPFMV, SPVG, SPVC, and SPV2); V2  =  plants derived from plant stock infected with SPCSV. Root length and number were transformed using log 10 and square root transformation, respectively, and Fisher's LSD test at the 0.05 probability level was used to test for statistical significance. The data are expressed as means ± SE from non-transformed data.

Representative images of LR development from each combination of nitrogen and virus treatment levels are shown in Fig. 3. The adventitious root derived from a virus-tested cutting grown in the presence of nitrogen has already manifested the initial stage of swelling in the proximal section (Fig. 3D), consistent with prior findings using this experimental system [8], [22]. The presence of a complex of viruses (SPFMV, SPVG, SPVC, and SPV2) was associated with 51% reduction in adventitious root number among plants grown without nitrogen when compared to the control treatment (Fig. 4A). The effect of virus treatment on first order LR attributes depended on the presence or absence of nitrogen (Figs. 4B–D). In the presence of nitrogen, only SPCSV-infected plants showed reductions in first order LR length, number, and density, which were decreased by 33%, 12%, and 11%, respectively, when compared to the controls. In the absence of nitrogen, virus tested and infected plants manifested significant reductions for all first order LR attributes that were measured. The use of infected plant material grown with or without nitrogen was associated with 57 to 96% reduction in second order LR length compared to the control treatment (Fig. 5A). The presence of virus and the lack of nitrogen were associated with 72 to 90% reduction in second order LR number relative to the control treatment (Fig. 5B). In general, optimum conditions for LR development were associated with the use of virus tested plants grown in nitrate sufficient conditions.

## Discussion

Other than demonstrating that certain strains of SPFMV can cause russet crack and determining effects of some viruses on storage root yield, little is known about how sweetpotato viruses affect the developing adventitious root system, especially during the critical storage root initiation period. Previous work has provided evidence that virus infected sweetpotato cuttings of the cultivar ‘Kokei No. 14′ had more adventitious roots but with lower weight when compared to virus-tested plants [29]. However, this study used a naturally infected storage root for generating planting material and the type of virus was not specified. This work [29] also did not consider lateral root development attributes nor did it relate virus effects to storage root initiation. The findings from the present study provide a framework for understanding how virus infection reduces sweetpotato yield, using a validated phenological model for storage root yield determination [5], given a scenario where vegetative cuttings are obtained from an infected source. First, the presence of a complex of viruses (SPFMV, SPVG, SPVC, and SPV2) was associated with the reduction of adventitious root number, regardless of the presence of nitrogen. Secondly, the presence of the phloem-restricted virus, SPCSV, was associated with the reduction in LR length, number, and density, previously associated with increased lignification in the stele, thereby preventing storage root initiation [8]. Both of these situations result in the net reduction of adventitious roots that can undergo storage root initiation and subsequent bulking, thereby negatively impacting yield potential. Previously, it has been shown that nitrogen deprivation resulted in reduced lateral root development which was associated with 44% and 55% reduction in uptake of phosphorus and potassium, respectively [22]. Thus, there is a concomitant effect associated with diminished nutrient foraging by the root system, with potential adverse effects on shoot growth and storage root bulking. Taken together, these findings parallel previous results from field studies that have documented the reduction of storage root yield in ‘Beauregard’, associated with the use of virus-infected propagating materials [3], [30].

It has been documented that auxin is the integrator of intrinsic and environmental signals that affect LR development [31] and that cytokinin has been shown to exert antagonistic effects [32], [33]. In the past, information on morphological, hormonal, and molecular characterizations of virus infection on roots have been limited [34], [35], but new findings are beginning to reveal the integrative role of auxin in mediating virus effects on root architecture. For example, *Arabidopsis* plants that were transformed to express the *Beet necrotic yellow vein* virus p25 protein showed abnormal root branching, accompanied by significant changes in the levels of auxin, jasmonic acid and ethylene precursor, ACC [24]. In another study, *Cucumber mosaic virus*-infected *Arabidopsis* root growth patterns were accompanied by significant changes in indole-3-acetic acid (IAA), *trans*-zeatin riboside and dihydrozeatin riboside [25]. In tomato (*Solanum lycopersicon*), infection by *Tomato aspermy* virus resulted in reduced LR development, which was accompanied by increased miR164 levels [23], a microRNA family previously associated with negatively regulating LR initiation in response to auxin by limiting NAC1 expression [36]. Parallel microarray experiments on shoots and roots from tomato infected with *Tomato spotted wilt virus* showed organ-specific responses although the virus was present in similar concentrations [34]. The study showed that in tomato shoots, genes related to defense and signal transduction were induced, while there was a general repression of genes related to primary and secondary metabolism as well as amino acid metabolism. In roots, genes related to biotic stimuli were induced while those related to abiotic stress were repressed. Currently, it is not known how sweetpotato viruses affect auxin or cytokinin levels or distribution in developing adventitious roots. It has been demonstrated that gene expression in leaves was altered in sweetpotato by virus infection as soon as 5 days after inoculation [37]. Single infections with SPFMV or SPCSV altered expression of 3 or 14 genes, respectively, as opposed to 200 genes in plants infected with both viruses [38]. In sweetpotatoes, the link between virus infection and root architecture marks a new research direction toward a better understanding of the relationship between virus infection and storage root yield. Some follow-up work might include the hormonal and molecular characterization of the mechanism of lateral root suppression by viruses, for enabling the development of tools and approaches that mitigate virus effect on sweetpotato productivity.

It has been suggested that root architecture may hold the key to the next green revolution [39], [40]. Root and tuber crops are second in importance to cereals as a global source of carbohydrates, with particularly high production potential in humid regions that are not suitable for cereal production [41]. Our findings in sweetpotato underscore the need to further investigate the effects of viruses on root architecture development in crops, especially those grown in low-input agricultural systems where virus diseases are a persistent threat.
